# The journey continues to make *CVIR Endovascular* THE open-access journal for all endovascular specialists: a few words from the new editor in chief

**DOI:** 10.1186/s42155-025-00636-w

**Published:** 2025-12-09

**Authors:** Robert A. Morgan

**Affiliations:** St George’s, University of London and St George’s University Hospitals NHS Foundation Trust, London, UK

I am delighted to take up the role of editor in chief for *CVIR Endovascular* following on from the excellent stewardship of the journal by Professor Jim Reekers. Jim Reekers took the journal from its inception 8 years ago to its status today with an impressive impact factor of 1.5. My aim as the new editor in chief is to take the journal even further to make *CVIR Endovascular* acknowledged as a key destination for endovascular specialists to publish their work.

I encourage all of you who are involved in endovascular work, whatever the vascular area, to submit your research work and ideas for research to *CVIR Endovascular* for publication. We are pleased to receive all types of manuscripts, including original scientific articles, technical notes, review articles, commentaries, case reports, and letters to the editor. Moreover, although the editorial office is based in Europe, we regard ourselves as a global endovascular journal and so encourage submissions from you, wherever you reside in the world.

I am always on the lookout for potential interesting submissions for this journal. If you have an idea for an article and you would like advice on whether this would be well received at *CVIR Endovascular*, I invite you to contact me at morgan@cvirendovascular.org to discuss your proposal with me.

One perceived drawback of open-access (OA) journals is the requirement for authors to pay the author publication charge (APC). However, OA publication means that any reader wherever they live can read your article without needing a paid subscription to access the journal. Most researchers and publishers opine that OA publishing is the future, and in time, OA will be the only way to publish scientific work. As this field evolves, many institutions throughout the world already have, or are, negotiating arrangements with publishers for their researchers to publish their work in OA journals without the need to pay the APC charge. Although not universal, this practice is progressively increasing year on year. You can check with your institution to ascertain whether the APC charge will be paid automatically. In addition, when your manuscript is accepted, our publisher, Springer, will automatically check if the APC charge needs to be paid or not.

I would like to highlight the hugely valued reviewers of our journal, without whom the journal would not be able to function. If you are reading this and you are not already a reviewer but would like to be one, please do not hesitate to contact the editorial office at *info@cvirendovascular.org* to enquire about becoming a reviewer. Once done, you can be added to the review database immediately.

I pleased to inform you that I have assembled a new team of section editors, each with a specialist endovascular focus to assist with the day-to-day management of the editorial process. There is also a new team of regional editors whose remit is to the promote the journal within their region and to work in concert with the section editors and the rest of the editorial team. Patrick Haage, whose professionalism and industry I have always thought highly of, has agreed to be the deputy editor in chief for this journal, for which I am grateful to his ongoing support.

*CVIR Endovascular* is a multidisciplinary endovascular journal, and in keeping with this, the new Editorial Board is comprised of endovascular specialists from all spheres of endovascular practice, including interventional radiologists, vascular surgeons, and angiologists.

CIRSE now has a family of journals, namely *CVIR Endovascular*, *CVIR Oncology*, and the original title, *CardioVascular and Interventional Radiology*. The CIRSE journal team shares an editorial office, and we will work closely with each other to harmonize the publishing sphere for minimally invasive therapy. In collaboration with the other two editors in chief, Tiago Bilhim and Thomas Helmberger, we will provide educational opportunities for aspiring publishers in the form of a series of manuscript writing webinars and on-site workshops at several international congresses throughout the year.

Finally, I would like to thank Martha Banegas and the team at *CVIR Endovascular* for all the work that they do to make the journal the quality publication that it has become and continues to be.

I am very much looking forward to the next few years as the editor in chief of your endovascular scientific journal.

*Robert A. Morgan*

*November 2025*

